# The cGAS-STING Pathway: A Promising Immunotherapy Target

**DOI:** 10.3389/fimmu.2021.795048

**Published:** 2021-12-09

**Authors:** Liang Ou, Ao Zhang, Yuxing Cheng, Ying Chen

**Affiliations:** Division of Pneumoconiosis, School of Public Health, China Medical University, Shenyang, China

**Keywords:** cGAS-STING signal pathway, macrophages, T lymphocytes, polarization, differentiation, immunotherapy

## Abstract

With the continuous development of immunotherapy, researchers have paid more attention to the specific immune regulatory mechanisms of various immune responses in different diseases. As a novel and vital innate immune signal pathway, the cGAS-STING signal pathway activated by nucleic acid substances, interplays with other immune responses, by which it participates in regulating cancer, autoimmune and inflammatory diseases, microbial and parasitic infectious diseases, and other diseases. With the exception of its role in innate immunity, the growing list of researches demonstrated expanding roles of the cGAS-STING signal pathway in bridging the innate immunity (macrophage polarization) with the adaptive immunity (T lymphocytes differentiation). Macrophages and T lymphocytes are the most representative cells of innate immunity and adaptive immunity, respectively. Their polarization or differentiation are involved in the pathogenesis and progression of various diseases. Here we mainly summarized recent advanced discoveries of how the cGAS-STING signal pathway regulated macrophages polarization and T lymphocytes differentiation in various diseases and vaccine applications, providing a promising direction for the development and clinical application of immunotherapeutic strategies for related diseases.

## Introduction

Invaded by exogenous or endogenous pathogens, the host immune system will be activated accordingly to resist harm and maintain homeostasis, which includes innate immunity and adaptive immunity. As the first line of host immune defense, innate immunity plays a critical role in recognizing extracellular and intracellular pathogens (1, 2). The major pathogens recognition receptors include Toll-like receptors (TLRs), C-type lectin receptors (CLRs), RIG-I-like receptors (RLRs), NOD-like receptors (NLRs), and cyclic guanosine monophosphate-adenosine monophosphate synthase (cGAS) (2).

**Figure 1:**
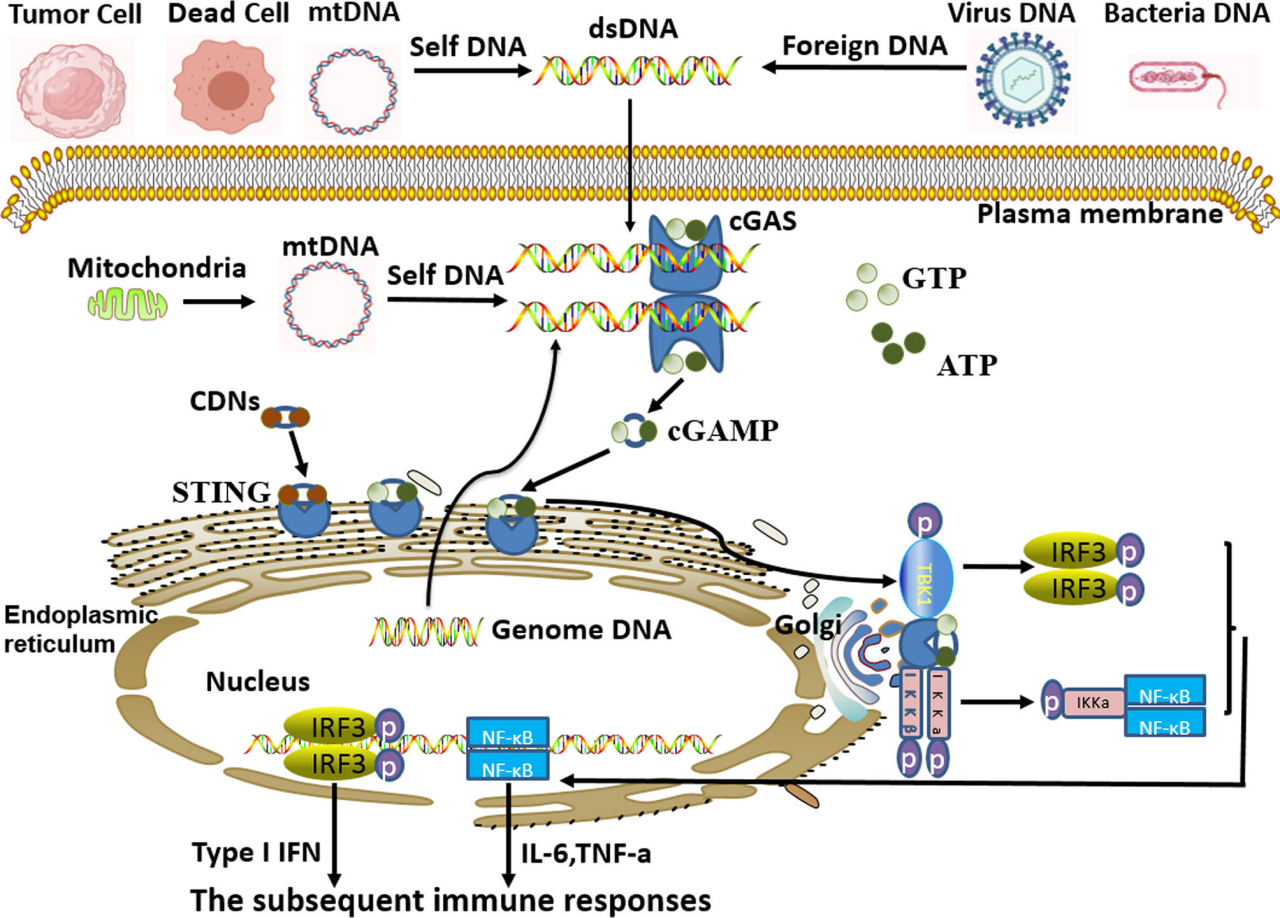
cGAS-STING signal pathway activated by cytoplasmic DNA. Free cytoplasmic DNA derived from self and foreign sources are specifically recognized by cyclic guanosine monophosphate-adenosine monophosphate synthase (cGAS). Then, activated cGAS induces the synthesis of cyclic guanosine monophosphate-adenosine monophosphate (cGAMP) with intracytoplasmic ATP and GTP as raw materials. As the secondary messenger, cGAMP or cyclic dinucleotides (CDNs) binds to endoplasmic-reticulum (ER) adapter protein STING (stimulating interferon gene), which causes a conformational change of STING and induces itself activation. Subsequently, activated STING migrates from ER to the Golgi apparatus. During this process, STNG could recruit and activate TBK1 kinase and IKK kinase, and in turn, activate the downstream IRF3 and NF-κB signal cascades, thus inducing the expression of type I interferon (type I IFN) and inflammatory factors to strengthen immune responses.

Among them, cGAS is the most concerning pathogens recognition receptor in recent years. In the process of pathogens gaining access to and proliferating inside the host cell, the pathogens’ DNA would release to and accumulate in the cytoplasm of the host cell. Simultaneously, the invading pathogens also destroy the host cell, causing its nuclear and mitochondrial DNA to be released into the cytoplasm. Then, all the free cytoplasmic DNA derived from self and foreign sources (tumor cells, dead cells, virus, microorganism) would be effectively recognized by cGAS. After recognition and binding of free cytoplasmic DNA to form a 2:2 complex in the cytoplasm (3, 4), there has been a conformational change in the active site of cGAS, which causes cGAS activation. Then the cyclic guanosine monophosphate-adenosine monophosphate (cGAMP) is synthesized by cGAS with intracytoplasmic ATP and GTP as raw materials. As the secondary messenger, cGAMP or cyclic dinucleotides (CDNs) binds to the adapter protein STING (stimulating interferon gene) anchored to the endoplasmic reticulum (ER), which generates a conformational change and activation of STING (4). Subsequently, cGAMP-bound STING migrates from ER to the Golgi apparatus. During the process of migration, STNG could recruit and activate TBK1 kinase and IKK kinase, and then activate the downstream IRF3 and NF-κB, thus inducing a robust expression of type I interferon (type I IFN) and a series of inflammatory factors to enhance immune responses (3, 5–12). (**Figure 1**)

STING is an adaptor protein that is expressed in macrophages, dendritic cells (DCs), and lymphocytes, as well as endothelial and epithelial cells (13). Macrophages, as major professional antigen-presenting cells (APCs), are best known for their ability to prime the host defense by engulfing foreign pathogens (14). The DNA contained in pathogens would activate the cGAS-STING signal pathway of macrophages, thereby producing many cytokines to cope with various stresses. In response to various changes and stimuli of cell internal environments, macrophages are activated and polarized into different phenotypes. In general, macrophages are roughly divided into two categories, one is called classically activated (or inflammatory) M1 macrophages, and the other is called selectively activated (or wound-healing) M2 macrophages (15–17). Recently, there is extensive research evidence emerging to suggest that the cGAS-STING signal pathway could significantly regulate macrophages polarization that is considered to be an important part of innate immunity, which may be used as a target for the immunotherapy of related diseases.

In addition, in response to intracellular environmental stress, the activation of the cGAS-STING signal pathway in APCs could produce a series of cytokines to promote the recruitment, maturation, activation, and differentiation of T lymphocytes, which then mediated subsequent adaptive immune responses (18–20). At present, we mainly divide T lymphocytes into two major groups. One is CD8^+^ T cells, which is particularly important for the protective immune responses against intracellular pathogens and tumors. During chronic infection or cancer, CD8^+^ T cells are exposed to persistent antigens and/or inflammatory signals. It could specifically recognize endogenous antigenic peptide-MHC I molecular complex, and then kill the infected cells or tumor cells *via* release of granules or induction of FasL-mediated apoptosis (21–23). The other is CD4^+^ T cells that could be further subdivided into different effector cell populations, including T helper type 1 (Th1), Th2, and Th17, which regulate diverse immune responses against pathogens; and regulate T (Treg) cell which plays an immunosuppressive function in order to prevent excessive or inappropriate inflammatory immune responses (24, 25). T lymphocytes differentiation is one of the most important aspects of adaptive immunity, which participates in the pathogenesis and progression of different diseases. Therefore, the accumulating studies indicate that the cGAS-STING signal pathway not only plays a powerful role in innate immunity but also plays a key regulatory role in adaptive immunity, serving as a bridge connecting innate immunity and adaptive immunity.

In this review, we will focus on the regulation and function of the cGAS-STING signal pathway in macrophages polarization (innate immunity) and T lymphocytes differentiation (adaptive immunity) in various diseases, so as to further clarify the pivotal role of the cGAS-STING signal pathway in linking innate immunity and adaptive immunity. In addition, we also used this as a basis to estimate the immunotherapeutic effects of the cGAS-STING signal pathway in the treatment of cancer, autoimmune and inflammatory diseases, microbial and parasitic infections, and the clinical application of vaccines, paving a promising direction for the development and clinical application of immunotherapeutic strategies for related diseases.

## Cancer

The host anti-tumor responses include innate immune interactions with cancer cells, including its recognition by innate cell populations (NK cells, NKT cells), and the recognition of dendritic cells and macrophages to damage-associated molecular patterns (DAMPs) derived from cancer cells. There is abundant evidence that the main driver of DAMPs in host anti-tumor immune responses is tumor-derived DNA (26). Unlike normal cells, there are often replete with free dsDNA in the cytoplasm of tumor cells. The dsDNA is derived from multiple sources including genomic, mitochondrial as well as exogenous origins, apoptotic cell, and transposable elements (27). Due to the abnormal phagocytic function of phagocytes or the degradation defects of phagocytic components, the tumor cell-derived DNA or the secondary messenger exist excessively in phagocytes, such as DCs and tumor-associated macrophages (TAMs), which then triggers the activation of the cGAS-STING signal pathway to induce host anti-tumor immune responses.

CD47 is a transmembrane protein with high expression in tumor-initiating cells, where elevated CD47 expression will inhibit the phagocytosis of phagocytes *via* binding to its receptor, signal regulatory protein α (SIRPα) which is expressed on phagocytes, thereby making the tumor cells escape the tumor immune surveillance (28–30). A study found that the treatment with antibody to CD47 could promote phagocytosis of phagocytes (DCs). Due to increased phagocytosis and the limited ability of phagocytes to clear engulfed contents, there was an abnormal increase and accumulation of tumor cell-derived DNA in the cytoplasm of DCs. Then, the excessive tumor cell-derived DNA could significantly activate the cGAS-STING signal pathway of DCs to strengthen antigen presentation and the activation and differentiation of CD8^+^ T cells to exert corresponding anti-tumor effects (31).

Under normal circumstances, cell apoptosis is in a state of immune immobilization (32, 33). Apoptotic cells can be effectively eliminated by phagocytes to prevent improper immune responses before the membrane integrity of apoptotic cells is destroyed and the cell contents are released. The phagocytosis of apoptotic cells is involved with multiple receptors such as the MER proto-oncogene tyrosine kinase (MerTK). MerTK, expressed in TAMs, belongs to a receptor tyrosine kinase family which can promote the dying and/or damaged cells to display the “eat me” signal *via* efferocytosis (34). Established a selected antibody specifically interacted with MerTK to treat the TAMs and the mice, the results showed that anti-MerTK antibody treatment caused defective efferocytosis of TAMs and the accumulation of apoptotic cells (35). The apoptotic cells (dying or damaged cells) that have not been effectively eliminated would release plentiful intracellular contents, including ATP and cGAMP (the secondary messenger), which was a result of the progressive loss of plasma membrane integrity. These released intracellular contents could be delivered to TAMs in the ATP-gated P2X7R channel to activate STING signal pathway-mediated type I IFN response. Type I IFN would promote the production of cytokines and chemokines to modulate antigen presentation function of TAMs and tumor-antigen-specific CD8^+^ T cells activation and differentiation to produce powerful anti-tumor immune effects.

In addition to abnormalities in phagocytosis, abnormalities in the degradation of apoptotic cells and the contents of engulfed cells could also induce the cGAS-STING signal pathway activation. LAP, LC3-associated phagocytosis, as non-classical autophagy, functions the process of engulfing particles, such as immune complexes, dying cells, apoptotic cells, and necrotic cells. After ingesting these components, the most critical role of LAP is to efficiently degrade the engulfed components to avoid producing accumulated cell components containing dsDNA and increased pro-inflammatory cytokines to over-activate related immune responses (36, 37). Recently, a study found that LAP impairment (deficiency in the special LAP functional protein, Rubicon) could enhance STING-dependent pro-inflammatory immune responses of TAMs, which was required for tumor resistance. Besides, it was intriguing that the enhanced STING-dependent immune responses were associated with increased activation of CD4^+^ and CD8^+^ T cells because the study found that depletion of T lymphocytes eliminated the LAP deficiency-induced anti-tumor effects (38). It’s worth thinking about that the damage to LAP resulted in obstacles to the elimination of apoptotic and dying cells. The abnormal clearance and degradation of apoptotic cells and dying cells could aggravate the formation and accumulation of autoantigens, including dsDNA, which was served as an important origin to activate the cGAS-STING signal pathway to mediate violent anti-tumor responses *via* regulating T lymphocytes differentiation. These findings indicated that the cGAS-STING signal pathway was a promising therapeutic target for the treatment of cancer.

Based on the critical role of the cGAS-STING signal pathway in the development of cancer, there are increasing studies have begun to focus on the treatment of tumors with agonists/drugs related to this pathway in recent years. A previous study found that manganese (Mn, Mn2^+^ in general cases) was served as a crucial element of host defense against DNA virus *via* enhancing the sensitivity of the DNA sensor cGAS and its downstream adaptor protein STING (39). So researchers speculated that Mn2^+^ might act as an effective agonist/drug to produce anti-tumor immune responses *via* activating the cGAS-STING signal pathway. Related results indicated Mn2^+^ treatment induced a significantly increased CD8^+^ and CD4^+^ TILs (tumor-infiltrating lymphocytes, TILs) in diverse tumor models to reduce tumor burdens. Using cGAS (cGAS−/−) and STING (Tmem173−/−) deficient mice, researchers confirmed that deletion of the pathway-related genes in mice caused complete loss of anti-tumor responses with no increased CD8^+^ or CD4^+^ TILs after Mn2^+^ administration (40). These data collectively suggested that Mn2^+^ promoted a T cell-mediated anti-tumor effect in a cGAS-STING-dependent way activating CD8^+^ and CD4^+^ T cells manner.

Other researchers found that the conventional STING agonist, DMXAA, could also induce the production of inflammatory cytokines (TNFα, IFN-γ, and IL-6) and chemokines (CXCL1, CXCL2, CXCL9, and CXCL10) to promote the migration, recruitment, differentiation, and expansion of macrophages and CD8^+^ T cells inside the tumor. These immune responses could effectively change tumor micro-architecture, affect the immune profile, and extend the survival of the Pancreatic ductal adenocarcinoma (PDA)-bearing mice tumor model, which was largely dependent on STING protein activation (41). Similarly, many other studies have also found CD8^+^ T cells-dependent anti-tumor responses are mediated by the cGAS-STING signal pathway (42–44). CD8^+^ T cells have long been considered as one of the most powerful anti-tumor effector cells (21, 22). Therefore, the above results indicated that the cGAS-STING pathway supplied significant anti-tumor effects, which might mainly depend on its regulation of activation and differentiation of CD8^+^ T cells.

Consistently, related research observed host anti-tumor CD8^+^ T cells responses loss when STING or IRF3 gene was knocked out in various tumor models (19). Moreover, STINGVAX, as a potent STING agonist, exerted a significant anti-tumor efficacy (quantitatively increased CD8^+^ TILs) in multiple tumor models. Using STING-mutant golden-ticket mice (C57BL/6J-Tmem173gt/J) to build an identical tumor model, researchers determined that functional STING was required for the anti-tumor efficacy of STINGVAX. Besides, STINGVAX efficacy in CD8^+^ T cells-depleted mice was diminished, which further indicated that CD8^+^ T cells were essential for the occurrence of STINGVAX-mediated anti-tumor efficacy (45). Similarly, in the breast cancer model, cGAMP (a common STING agonist) treatment activated the STING signal pathway to produce anti-tumor effects, which were impaired by using the anti-CD8 antibody to deplete CD8^+^ T cells of the tumor microenvironment (46). Other studies have also found that the cGAS-STING signal pathway could regulate CD8^+^ T cells differentiation to produce anti-tumor responses, including hepatocellular carcinoma, breast cancer, melanoma, colon cancer, and lung cancer (31, 47–52). Besides, CD8^+^ T cells depletion significantly decreased tumor regression with CDNs (a common STING agonist) treatment (53). These results further confirmed that the potent anti-tumor effect of the activated cGAS-STING pathway was indeed derived from its regulation of CD8^+^ T cells activation and differentiation.

Nevertheless, the tumor regression of CDNs was only partially abrogated with CD8^+^ T cells depletion, indicating that there were other cell types participating in the anti-tumor response of CDNs. What’s more, researchers found that CDNs treatment skewed the T cell repertoire toward the Th1 type with increased levels of IFN-γ (53). Consistent with this result, intra-tumor injection of STING agonist could not only induce strong CD8^+^ T cells responses but also induce Th1 differentiation or reduce the suppressive Foxp3^+^ Treg populations to a large extent to exert anti-tumor effects (41, 54, 55). All above results suggested the cGAS-STING pathway, as a cytosolic DNA-sensing pathway, played the potential contribution of anti-tumor efficiency through mediating the recruitment, activation, and differentiation of CD4^+^ and CD8^+^ T cells, especially the CD8^+^ T cells.

In addition to T lymphocytes, the anti-tumor effect of the activated cGAS-STING signal pathway is also closely related to TAMs. TAMs, as an important type of tumor-infiltrating immune cells, are involved with tumor growth and metastasis by influencing tumor immune surveillance. M1 and M2 macrophages are two extreme functional continuums of the complete polarization of anti-tumor TAMs and immunosuppressive TAMs (a class of tumor-promoting cells), respectively (56, 57). TAMs are the main host cells for the occurrence of the cGAS-STING signal pathway in cancer. A growing body of studies has found that the activation of the cGAS-STING signal pathway in TAMs is related to TAMs polarization that is necessary for bridging innate and adaptive immune responses, thereby regulating the tumor progression. For example, the absence of LAP would activate the STING signal pathway *via* the produced autoantigens (dsDNA) to induce pro-inflammatory gene expression (IL-6, TNF-α), polarizing TAMs into M1 macrophages and promoting the activation and differentiation of T cells to restrict tumor growth (38).

Considering the key role of the cGAS-STING signal pathway and related TAMs in anti-tumor immunity, many studies have explored whether the related STING agonists could exert strong anti-tumor effects *via* regulating TAMs polarization. Researchers found that DMXAA efficiently repolarized M2-type bone marrow-derived macrophages into M1-type macrophages, characterized by increased levels of IL-6, TNFα, and several costimulatory molecules that promoted CD8^+^ T cells recruitment, activation, and differentiation. The two kinds of cells synergistically exerted tremendous anti-tumor effects, which were indeed dependent on STING activation (41). The previous results demonstrated that TAMs played a necessary role in the recruitment and differentiation of T cells, which might be one of the mechanisms by which the cGAS-STING signal pathway exerted powerful anti-tumor effects. And the same results were found in mouse non-small cell lung cancer (58).

In different tumor models, activation of the cGAS-STING signal pathway was found to be significantly associated with infiltration of macrophages, CD4^+^ T cells, CD8^+^ T cells, B cells, and dendritic cells, as well as their immune markers (47, 49). These results further demonstrated that the cGAS-STING signal pathway played an important role in recruiting the related immune effector cells in the process of cancer. Consistently, injecting the STING agonist, cGAMP, into the tumor exhibited effective anti-tumor responses by recruiting and activating M1 phenotype TAMs in a STING-dependent manner *via* producing TNF-α, type I IFN, and T-cell-attracting chemokines to mediate various immune responses. Depleting the TAMs would decrease the anti-tumor effects of cGAMP (46, 59). Besides, liposomal nanoparticle-delivered cGAMP (cGAMP-NP) could directly activate STING of macrophages, repolarize tumor-promoting M2-type macrophages into M1-type macrophages, enhance MHC class molecules, or co-stimulatory molecules, and then induce the differentiation of CD4^+^ and CD8^+^ T cells to produce intense anti-tumor responses (48). The above results revealed that the production of activated M1 TAMs was important for the occurrence of effective anti-tumor immune responses, which were dependent on the activation of the cGAS-STING signal pathway.

DMXAA and cGAMP are only effective agonists for murine STING, but they cannot activate human STING. Here, some researchers found that α-mangostin was a potent agonist of human STING, and could also activate murine STING to a lesser extent. Consistent with the functions of DMXAA and cGAMP, a-mangostin could also increase M1-type markers, decrease M2-type markers in a dose-dependent manner, and repolarize M2 TAMs into M1 TAMs in a STING signal pathway manner, accounting for the anti-tumor activities (57). The above studies demonstrated that the STING signal pathway was strongly involved in TAMs polarization in humans and murine, which was mainly manifested as the polarization of TAMs into M1 phenotype to defense tumor. Also, this laid the foundation and highlights the prospect for the development of drugs with characteristics of STING agonists in cancer treatment.

Based on these findings, many preclinical studies have begun to use direct pharmacological stimulation of the cGAS-STING signal pathway as a new way to treat cancer. At the same time, whether the cGAS-STING signal pathway is related to radiotherapy and chemotherapy, and whether it can be combined to play an effective anti-tumor effect, has attracted more and more attention from researchers. In different studies, researchers have found that radiotherapy could cause DNA damage, resulting in the accumulation of dsDNA, which could enter DCs through various transfer pathways to activate the cGAS-STING signal pathway. The activated cGAS-STING signal pathway would induce interferon (IFN) production to promote DCs maturation and activation, which mediated CD8^+^ T cells activation to provide tumor regression (43, 60–62). Of course, the activation of the cGAS-STING signal pathway is also determined by the dose and frequency of radiotherapy. Radiation doses above 12-18 Gy could also activate the DNA exonuclease Trex-1 which degrades the accumulated dsDNA to reduce tumor immunogenicity and promote tumor immune escape (62). Appropriate radiation dose could effectively activate the cGAS-STING signal pathway to stimulate the production of IFN. IFN could induce the recruitment and activation of DCs which is essential for priming of CD8^+^ T cells to mediate tumor rejection (62). If the radiation dose is not sufficient to support cancer cells producing enough IFN to resist the tumor, what we can do is to insert STING agonists into tumors to induce IFN to enhance the tumor resistance response (43). Similarly, one study found that nanoparticle-cGAMP inhalation plus radiotherapy could induce regression of lung metastases in a STING signal pathway mediated effector CD8^+^ T cells manner (63). The above results suggested that the anti-tumor immunity generated by radiotherapy alone or the combination of radiotherapy and STING agonists depended on the regulation of CD8^+^ T cells activation and differentiation by the cGAS-STING signal pathway, which also provided a direction for the combined use of the cGAS-STING signal pathway agonists and radiotherapy for tumor treatment.

In addition, chemotherapy is also one of the most conventional ways to eliminate tumors. The cGAS-STING signal pathway was found to play an important role in the inhibition of tumor growth by paclitaxel (64). Cisplatin is a chemotherapy drug that could induce DNA damage, which could enhance the STING signal pathway mediated-tumor elimination, characterized by increasing antigen presentation and T-cells infiltration (65). Moreover, numerous studies demonstrated that the combination of cisplatin and IFN could effectively inhibit tumor growth and prolong the survival time of mice more than cisplatin alone (66–68). 5-fluorouracil (5-FU) is an important chemotherapeutic drug that mainly interferes with DNA synthesis, although its use has some side effects. The researchers found that cGAMP could further enhance the anti-tumor immune response of 5-FU and effectively reduce the cytotoxicity of 5-FU when used in combination with cGAMP and 5-FU. The possible mechanism might be that cGAMP activated the STING signal pathway, which could induce the production of cytokines to activate the DCs. Then, the activated DCs would induce the cross-priming of CD8^+^ T cells to produce an anti-tumor response. In addition, the activated dendritic cells might be able to reduce 5-FU-induced cytotoxicity (69). These results indicated that chemotherapeutic drugs might also exert their anti-tumor effects by activating the cGAS-STING signal pathway, and the combination of chemotherapy and the cGAS-STING signal pathway agonists would also be a promising research field for tumor treatment.

The above experiment results suggested that the activation of the cGAS-STING signal pathway could produce significant anti-tumor effects *via* regulating T lymphocytes differentiation and TAMs polarization. On the contrary, some researchers found that the activated cGAS-STING signal pathway could also enhance the expression of several counter-regulatory immune parameters to a certain extent, including programmed death-ligand 1 (PD-L1), CD4^+^ Foxp3^+^ regulatory T cells, and indoleamine 2,3-dioxygenase (IDO). And all of the related factors could effectively inhibit host anti-tumor immunity *via* inhibiting the activation and differentiation of the effector T cells or promoting the polarization of M2 TAMs (70–73). Consistently, some research results showed that tumor cell-derived particles (membrane molecules, nuclear histones, caspases, microRNAs, and DNA) polarized TAMs into M2 phenotype and promoted the apoptosis of M1 TAMs, thus promoting tumor growth and metabolism, which were regulated by cGAS/STING/TBK1/STAT6 signal pathway (74). In addition, relevant studies have also found that activation of the cGAS-STING signal pathway has a positive effect on tumor growth, proliferation, and metastasis (75, 76). Chromosomal instability caused micronuclei damage and induced the production of cytoplasmic dsDNA, which could effectively activate the cGAS-STING signal pathway to promote tumor metastasis (77). A recent study appears to have discovered the immune mechanism by which tumors escape immune surveillance. The ectonucleotidase ENPP1, as a negative regulator of cGAMP, is upregulated in tumor cells with chromosomal instability. It selectively degrades the extracellular cGAMP to produce the immune suppressor, adenosine, which could promote the migration and metastasis of the tumor. Meanwhile, the cytoplasmic dsDNA induced by chromosomal instability could promote tumor metastasis in a manner dependent on the cGAS-STING signal pathway of tumor cells. Conversely, ENPP1 loss or inhibition could effectively increase extracellular levels of cGAMP. Accumulating cGAMP would be transported to host cells through the paracrine pathway to activate the STING signal pathway of host cells, exerting a powerful anti-tumor immune response, characterized by increasing CD8^+^ T-cells density and suppressing tumor metastasis in a manner dependent on host STING (78). These results indicated that under certain conditions, the activation of the cGAS-STING signal pathway might produce anti-tumor effects and pro-tumor effects through different regulatory processes, respectively.

Based on current research evidence, we have demonstrated that the cGAS-STING signal pathway mainly promotes the polarization of TAMs into M1 phenotype to secrete inflammatory factors and chemokines to recruit and activate T lymphocytes, which could promote the differentiation of CD4^+^ and CD8^+^ T cells to defend against cancer, linking innate immunity and adaptive immunity. All of these indicated a prospect of tumor immunotherapy using the cGAS-STING signal pathway-related agonists alone or in combination with radiotherapy and chemotherapy. Given the diversity and complexity of tumor cell immune processes, the mechanism of the cGAS-STING pathway on tumors is still a field worthy of further exploration.

## Autoimmune and Inflammatory Diseases

Activation of the cGAS-STING signal pathway triggered by nucleic acids is involved in the pathogenesis of various autoimmune diseases, including Aicardi–Goutieres Syndrome (AGS), systemic lupus erythematosus (SLE), familial chilblain lupus (79–82). In AGS, STING-dependent type I IFN responses could drive T lymphocytes to mediate cellular inflammation and autoantibody responses, including CD4^+^ and CD8^+^ T cells (83). In type I diabetes, researchers found that activated STING could modulate T cells immunity. The production of type I IFN dependent on STING could induce the production of IDO, promoting Treg cells differentiation and thus exerting immunosuppressive effects to attenuate type I diabetes progression (84). IDO could also inhibit Th1 cells proliferation and induce Treg cells differentiation in a cGAS-STING signal pathway manner, thereby attenuating the joint injury inflammation and destructive immunity of rheumatoid arthritis (85). The above studies indicated that the cGAS-STING pathway might also participate in the pathogenesis of autoimmune diseases by regulating T cell differentiation, but the research about these was not perfect, which was a direction worthy of our active exploration in the future.

What’s similar is that STING activation could cause numerous inflammatory diseases, owing to the continuous production of inflammatory cytokines. A representative example is that acquired mutations of the STING gene (STING N154S in humans and STING N153S in mice) cause STING–associated vasculopathy with onset in infancy (SAVI), characterized by vasculopathy, ulcerative skin lesions, and pulmonary fibrosis, spontaneous colitis (86–91). In the SAVI model, STING activation led to a cell-intrinsic T-cells defect (CD4^+^ and CD8^+^ T cells) *via* regulating the differentiation of progenitor cells and the lifespan of mature T cells (88, 92). Subsequently, the impaired proliferation of T cells and other immune cells would cause severe innate and adaptive immunodeficiency to exacerbate the severity of SAVI. Recent studies have suggested that the reason for the T-cells deficiency caused by STING activation may be STING activation disrupt the calcium homeostasis or the migration of progenitors to the thymus (88, 89, 92, 93). These findings suggested that STING was a critical factor in the occurrence and development of the SAVI *via* regulating the proliferation and differentiation of the T cells. Future work needs to further clarify the specific immunomodulatory mechanism of STING in SAVI, so as to explore and develop relevant immunotherapy methods.

Like SAVI, COPA syndrome is a monogenic autoinflammatory disease caused by the mutation of the Coatomer protein complex subunit alpha (COPA) gene, characterized by arthritis and lung disease. In the mouse model of COPA syndrome, mutant COPA impaired the thymic selection of T cells, characterized by an increase of IFN-γ and IL-17A-secreting CD4^+^ T cells and IFN-γ-secreting CD8^+^ T cells, and a decrease of regulatory T cells in peripheral tissues. These immune disorders would cause inflammation and tissue damage. COPA could maintain immune homeostasis by mediating the retrieval of STING from the Golgi to ER. The defects of COPA caused STING to maintain protein polymerization and become spontaneously activated at the Golgi. Activated STING stimulated type I IFN-driven inflammation in COPA model mice that was rescued in STING-deficient animals. Also, STING deficiency reversed the significant increase of activated effector T cells caused by mutant COPA (12, 94, 95). These results suggested that STING played a key role in COPA mutation-induced immune disorders, mainly by promoting the differentiation of effector T cells, which indicated that we could develop effective STING pathway inhibitors to treat COPA symptoms.

As we describe in the SAVI section, the constitutive activation of STING in N153s mice can also result in spontaneous colitis that is also an important manifestation of SAVI. STING activation and accumulation predominantly occurred in intestinal myeloid cells including macrophages and monocytes in colitis. The researchers found that the c-di-GMP triggered the ubiquitination and stabilization of STING to induce a significant increase of IFN-γ-producing Th1 cells and reduction of Treg cells, promoting the progression of colitis (96), which proved that STING could regulate the differentiation of T cells in the SAVI model to affect the process of the disease once again. Similarly, in the DSS-induced experimental colitis model, STING protein levels were significantly increased in M1 phenotype bone marrow-derived macrophages (BMDMs) or the PMA-differentiated human THP-1-derived macrophages, which predicted that M1 polarized macrophages expressed increasing sensitivity to CDNs. Consistent with the inference, subsequent experiments confirmed that CDNs could induce STING activation to exacerbate the severity of DSS-induced colitis, including the colonic damage and inflammation, through inducing the polarization or repolarization of M1 macrophages from both M0 and M2 macrophages (97). Therefore, STING as a sentinel of intestinal homeostasis could be served as an important target to explore effective strategies to treat related intestinal diseases.

Analogously, STING expression was increased in liver tissues from patients with non-alcoholic fatty liver disease (NAFLD) or the mice with HFD (high-fat diet)-induced steatosis. And STING knockout exhibited increased expression of macrophage alternative (M2) activation, and decreased expression or release of pro-inflammatory cytokines, the markers of M1 phenotype macrophages, to alleviate the disease severity (98). These discoveries indicated that STING activation would aggravate the severity of related liver inflammatory disease by promoting M1 macrophages polarization.

In addition, STING activation would worsen acute pancreatitis severity *via* macrophage sensing of DNA released from dying acinar cells (99). On the contrary, researchers found that STING activation could alleviate the inflammation and fibrosis of chronic pancreatitis. The absence of the cGAS-STING signal pathway would enhance Th17 differentiation to produce IL-17A. IL-17RA expressed in pancreatic stellate cells (PSCs) responded to IL-17A to promote the progression of inflammation and fibrosis *via* activating downstream ERK1/2 (100). These results suggested that the cGAS-STING signal pathway played a negative role in the pathogenesis of chronic pancreatitis by regulating Th17 differentiation. The above results suggested that the cGAS-STING signal pathway might play distinct immunomodulatory roles in different stages of inflammatory diseases, which required further study.

Current experimental results suggested that there were different dominant cells in various autoimmune and inflammatory diseases. Also, it is worth affirming that in-depth exploration of the role of the cGAS-STING pathway and its related drugs in autoimmune and inflammatory diseases is expected to provide a new approach for the treatment of related clinical diseases.

## Microbial and Parasitic Infections

When the host is infected by microorganisms and parasites, recognizing the nucleic acids and CDNs released by them, the cGAS-STING signal pathway of host cells will be activated and then induce striking immune responses to control and defend against the pathogen invasion. Therefore, the study of immune processes in this research field may contribute to the prevention and treatment of related pathogenic microbial and parasitic infections.

Streptococcus pyogenes, as an important Gram-positive human pathogen, could express a wide spectrum of clinical manifestations, including mild skin or mucosal surfaces infections, necrotizing fasciitis, and toxic shock syndrome (101, 102). Researchers identified that Streptococcus pyogenes could effectively induce the production of type I IFN in a STING-dependent manner (103). The possible mechanism was that Streptococcus pyogenes could release c-di-AMP to activate STING directly. Then, the type I IFN signal would promote the immunosuppressive cytokine IL-10-producing M2 macrophages polarization to curb the inflammatory responses of infection, which was dependent on the activation of the STING not cGAS in a DNA-independent manner. Similarly, the STING signal pathway (activated by c-di-GMP or cGAMP) has been found to play an important role in Streptococcus pneumoniae, Bordetella pertussis, and bacilli-infected diseases. The difference lies in that STING mainly resists pathogen invasion to the host by inducing Th (Th1/2/17) cells and CD8^+^ T cells differentiation during the infection of these pathogens, rather than affecting the polarization state of macrophages (104–106). Mycobacterium bovis could promote maturation and activation of bone-marrow derived-dendritic cells (BMDCs) in a cGAS/STING/TBK1/IRF3 signal pathway-dependent manner. Then the activated BMDCs were able to release plentiful cytokines to promote the CD4^+^ T cells proliferation and differentiation, effectively resisting the invasion of Mycobacterium bovis (18). Trypanosoma cruzi infection is a common parasitic infection. As an agonist for STING, c-di-GMP could produce positive protective effects by promoting Th1/2/17 cells and CD8^+^ T cells responses to defend against the Trypanosoma cruzi infection, as described in the section about vaccine application, but will not be described in detail here (107–109). Therefore, the above evidence indicated that the activation of the STING signal pathway could effectively resist the invasion of some microbial pathogens to the host by regulating macrophages polarization or T lymphocytes differentiation.

In contrast, other studies found that STING could intensify the invasion ability of some pathogens to the host. Mycobacterium tuberculosis (Mtb), as a very common infectious bacteria, led to lung cells lesions, including macrophages and B lymphocytes (110–112). B lymphocytes not only played the role of producing antibodies but also could significantly influence the process of infectious or non-infectious diseases through an antibody-independent mechanism. Researchers found that the expression of type I IFN in B cells purified from the lungs and spleen of Mtb-infected mice also involved the innate receptor STING. Treating macrophages with the supernatant from Mtb or c-di-AMP-stimulated B cells, the results suggested that macrophages exhibited an enhanced expression of regulatory/anti-inflammatory molecules PD-L1 and IL-10, which confirmed that STING-triggered type I IFN was involved in macrophages polarization toward a regulatory/anti-inflammatory phenotype, M2 macrophages, during Mtb infection, enhancing the invasion ability of Mtb (113). Similarly, others found that the activation of the cGAS-STING signal pathway induced by pre-exposed silica would be furtherly enhanced *via* recognizing the Mtb DNA. These enhanced immune responses might exacerbate IFN dependent type 2 immunity manifested by anti-inflammatory M2 macrophages polarization and Th2 lymphocytes differentiation to strengthen Mtb invasion ability (114). Interestingly, we could find that no matter in the case of infection of Streptococcus pyogenes or Mtb, the STING signal pathway promoted the polarization of M2 macrophages through the production of IFN, and then participated in the disease process.

Based on the above research findings, we have found that the cGAS-STING signal pathway is a double-edged sword, which exacts distinct effects *via* inducing different macrophages polarization and T lymphocytes differentiation in the pathogenesis and progression of diverse microbial and parasitic infectious diseases, some of which are detrimental and some of which are protective. What we need to do is to make use of the different functions it plays to effectively prevent and treat related diseases.

## Other Diseases

Bone metabolism is important in osteoporosis, joint disease, rheumatoid disease, and other diseases. Bone homeostasis is maintained through bone-forming osteoblasts and bone-resorbing osteoclasts derived from monocyte/macrophage lineage. CDNs, as commensal bacteria-derived second messengers, are expressed at high levels in the gut. It could deliver into the blood *via* extracellular vesicles and express stably in the serum or tissues (115). CDNs have an important immunomodulatory efficiency through inducing STING signal pathway activation to express type I IFN in macrophages. Following that, researchers speculated whether CDNs could be absorbed into the bone marrow to influence bone metabolism by regulating osteoclasts differentiation. Related results proved that CDNs could indeed be absorbed into the bone marrow, and then inhibit marrow-derived macrophages (BMMs)-induced osteoclasts differentiation in a STING-mediated signal pathway-dependent manner (115) because the inhibition of osteoclast differentiation and the phosphorylation of TBK1 and IRF3 (a representative feature of STING activation) caused by CDNs were repaired in STING knockdown BMMs-induced osteoclasts. Moreover, the inhibitory effects of CDNs on osteoclasts differentiation were also diminished in osteoclasts treated with the antibody blocking IFN-α/β receptor (IFNAR) or derived from IFNAR1-/- mice (115), which indicated that type I IFN was the crucial negative regulator of osteoclastogenesis. In a nutshell, the above results suggested that CDNs could inhibit osteoclasts differentiation by activating the STING-IFN signal pathway to inhibit osteoporosis. In contrast, another study found that the STING signal pathway affected osteoclasts’ differentiation by regulating other downstream signal cascades. NF-κB signal pathway, as an important STING-mediated downstream signal cascade, also participated in the progression of osteoclastogenesis. Researchers found that RTA-408, as a novel synthetic oleanane triterpenoid compound, could inhibit osteoclasts differentiation *via* constraining STING-mediated NF-κB signal, characterized with suppression of the STING gene expression and the phosphorylation of IκBα and P65. It was important to note that RTA-408 did not have any effect on the STING-IFN-β signal. And STING overexpression treatment could rescue the suppressive effects of RTA-408 on NF-κB signal and osteoclastogenesis (116). Collectively, what could be proved was that RTA-408 inhibited osteoclasts differentiation *via* effecting STING mediated NF-κB signal.

The experimental results about osteoporosis have indicated that there are two main downstream signal cascades of the cGAS-STING signal pathway, one is the IFN pathway and the other is the NF-κB pathway, which plays completely distinct effects in regulating the differentiation of osteoclasts (an important Tissue-Resident Macrophages). Under immune homeostasis conditions, they maintain a state of immune balance between them. Once immune homeostasis is broken, the trade-off between them determines the final immune endpoint, which could cause completely different immune effects. However, the explicit regulatory mechanism is not clear yet, which needs further study and exploration.

In addition, in the traumatic brain injury (TBI) diseases model, researchers found that neuronal-endoplasmic reticulum stress could induce the activation of the neurons-STING signal pathway, which could release IFN-β to enforce original microglia cells (an important Tissue-Resident Macrophages) activation and polarize them into M1 macrophages to release pro-inflammatory chemokines. The chemokines would promote Th1 cells differentiation to cause and aggravate brain injury (117). Consistently, the danger-associated molecular patterns (DAMPs) produced in the cell or mouse ischemic stroke (IS) model could strongly activate the cGAS-STING signal pathway to trigger microglia M1 polarization, expressing high levels of pro-inflammatory cytokines (TNF-α, IL-1β, IL-12) and inducible NO. Using siRNA technology to knock down cGAS, researchers found that knockdown of cGAS could significantly decrease microglial M1 polarization and increase microglial M2 polarization, attenuating microglia-mediated inflammation and neurological impairment (118). Interestingly, similar results were found in myocardial infarction (MI). Ischemic myocardial injury caused massive cell injury and necrosis and consequent release of both nuclear and mitochondrial DNA. The released DNA would activate the cGAS-STING signal pathway to induce the M1 (an impaired phenotype) polarization which could aggravate the tissue damage. cGAS loss-of-function eliminated the production of M1 macrophages and related inflammatory programs and promoted the M2 (a reparative phenotype) polarization. The transformation of macrophages could improve heart failure (119).

Data above all showed that the cGAS-STING signal pathway had a certain regulatory effect on macrophages polarization and T lymphocytes differentiation in osteoporosis, TBI, IS, and MI. Inhibiting the activation of this pathway could greatly reduce the severity of the associated diseases. Although the research of immunotherapy for cardiovascular and central nervous system diseases is still in its infancy, it is an area full of potential and promise. These different immune regulatory mechanisms could be served as an efficient therapeutic target for us to protect and defend the related diseases.

## Application of Vaccines

For the treatment of diverse diseases, including cancer, AIDS, tuberculosis, anthrax, pertussis, the continuous improvement and development of effective vaccines are essential. Among them, in-depth research and analysis of the suitable vaccine adjuvants are of great significance to the rational strengthening of vaccines immunogenicity. Chitosan, a potent and powerful vaccine adjuvant, promotes the intracellular release of DNA to enhance antigen-specific Th1 immune responses in a cGAS-STING-IFN signal pathway-dependent manner, which induces strong cellular immunity (120, 121). This proved that the cGAS-STING signal pathway played a key target role in the treatment of various diseases *via* vaccine adjuvants.

In cancer immunotherapy research, the use of cancer vaccines is evolving. The irradiation treatment could induce enhanced expression of reactive oxygen species (ROS) and oxidized mtDNA in tumor cells. As a danger-associated molecular pattern (DAMP) or adjuvant, oxidized mtDNA would gain access to the cytosol of DCs and activate the STING signal pathway, which subsequently presented irradiated tumor cell-derived antigens to promote the proliferation and differentiation of CD8^+^ T cells eliciting strong anti-tumor immunity of the irradiated immunogenic cancer cell vaccine (122). Before that, we have summarized that Mn2^+^ could serve as an effective drug to produce anti-tumor immune responses *via* activating the cGAS-STING signal pathway dependent on dsDNA (40). Further study found that Mn2^+^ could directly activate cGAS independent of dsDNA to facilitate the antigen uptake and presentation of APCs, thereby inducing the production of antibody and the proliferation and activation of CD4^+^/CD8^+^ T cells to strengthen humoral and cellular immune responses (123), indicating that Mn2^+^ could serve as a promising adjuvant against tumors. Similarly, earlier in this article, we have reviewed that STINGVAX (a STING agonist), as the first designed cancer vaccine containing granulocyte-macrophage colony-stimulating factor (GM-CSF) and CDNs, exerted a significant anti-tumor efficacy in multiple therapeutic models *via* increasing CD8^+^ TILs infiltration (45). All the evidence showed a crucial role of STING agonists as a cancer vaccine adjuvant in immunotherapy for cancer.

Also, injecting STING agonists into the skin as a vaccine adjuvant could activate Th1 immune responses, which was characterized by high levels of Th1 cytokines (IFN-γ and IL-2), effectively fighting against allergic skin inflammation (124). Besides, many studies have confirmed that diverse STING agonists, including cGAMP, c-di-AMP, and c-di-GMP, could be used as potent adjuvants to enhance vaccines efficacy. cGAMP could activate and enhance CD4^+^ T cells (Th1/Th2/Th17) and CD8^+^ T cells responses against the virus invasion as a powerful adjuvant (125–131). c-di-AMP and c-di-GMP also showed good prospects in the application of vaccine adjuvants (107–109, 132–135). c-di-AMP could induce strong Th1/17 immune responses, resisting Trypanosoma cruzi infection and Mycobacterium tuberculosis infection (107–109, 133). Similarly, the related study found that c-di-GMP could produce strong CD4^+^ T cells and CD8^+^ T cells responses, which were of great significance for the immunotherapy of related infectious diseases in a STING–NF-κB–TNF-α pathway manner, including H5N1 influenza, Staphylococcus aureus, and Streptococcus pneumoniae (134, 135).

The above results indicated that STING agonists, as vaccine adjuvants, could synergize with the vaccine to produce striking cellular and humoral immune responses to enhance vaccine efficacy in a cGAS-STING signal pathway-dependent T lymphocytes proliferation and differentiation manner, which could significantly resist the tumor and foreign pathogens invasion.

## Conclusions

**Figure 2:**
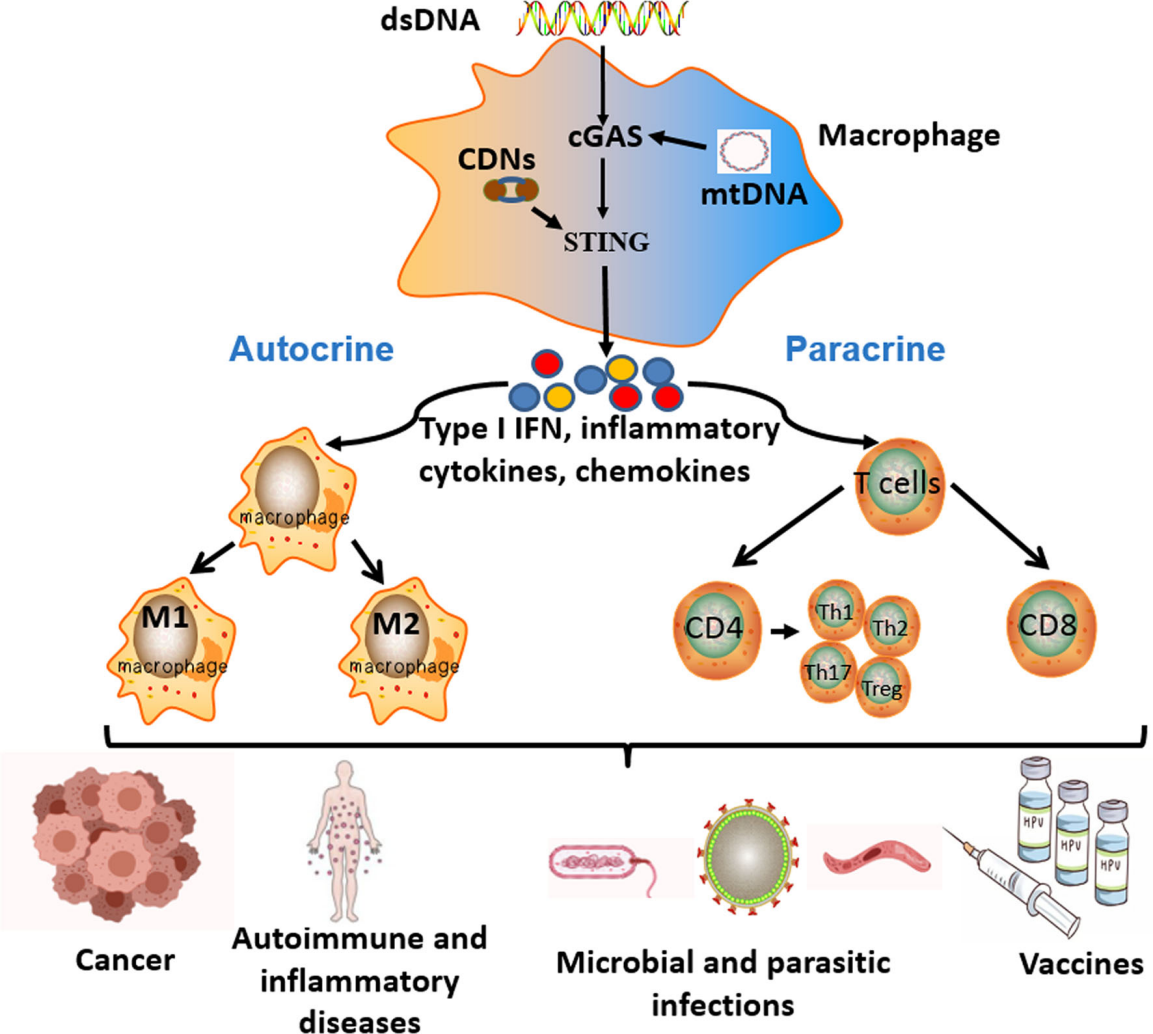
The key roles and effects of the cGAS-STING signal pathway in different diseases. Free cytoplasmic dsDNA or CDNs could activate the cGAS-STING signal pathway of antigen-presenting cells (APCs). Activation of the cGAS-STING signal pathway induces a series of immune cascades to produce diverse products, including type I IFN, inflammatory cytokines, and chemokines. These products have significant influences on the host immune microenvironment in both autocrine and paracrine ways. In an autocrine way, it could promote the maturation, activation, and polarization of macrophages. In a paracrine way, the different cytokines produced by APCs could recruit T lymphocytes and promote their proliferation and differentiation. All the above immune responses participate in the pathogenesis and progression of various diseases, as well as the effective process of vaccines.

In this review, we summarized that in various diseases, including cancer, autoimmune and inflammatory diseases, microbial and parasitic infectious diseases, and other diseases, free dsDNA or CDNs derived from cancer cells, apoptotic or necrotic cells and pathogenic microorganisms could activate the cGAS-STING signal pathway of APCs to induce a series of immune cascades, producing various products such as type I IFN, pro-inflammatory cytokines and chemokines. These products had significant impacts on the host cellular microenvironment in both autocrine and paracrine manners. In an autocrine way, it could promote the maturation and polarization of macrophages and enhance their ability of antigen presentation and cytokine secretion. In a paracrine way, the type I IFN, pro-inflammatory cytokines, and chemokines produced by activated APCs could recruit T lymphocytes and promote their proliferation and differentiation. All these immune processes played an important regulatory role in the onset and progression of various diseases. Similarly, STING agonists (such as CDNs) as vaccine adjuvants could enhance vaccine-induced immune effects *via* activating the STING signal pathway, including the APCs-mediated innate immune responses and the subsequent different effector T lymphocytes-mediated adaptive immune responses. The enhanced cellular and humoral immune responses of vaccines could significantly resist the invasion of the host by foreign pathogens. (**Figure 2**)

However, in the process of summarizing these findings, we also found that there were still some doubts and contradictions regarding the regulation and function of the cGAS-STING signal pathway on macrophages polarization and T lymphocytes differentiation. Although most studies on tumors and inflammatory diseases have shown that cGAS-STING signal pathway activation could induce the M1 polarization of macrophages, some studies on microbial infections have found that activated cGAS-STING signal pathway could also promote the M2 polarization of macrophages. Related studies found that the highly expressed type I IFN triggered by STING activation mediated the M2 polarization of macrophages, characterized by enhanced expression of regulatory/anti-inflammatory molecules PD-L1 and IL-10 in macrophages (113, 123). Similarly, another study found that Mycobacterium tuberculosis DNA could further enhance the activation of the cGAS-STING signal pathway induced by pre-exposed silica to promote the type I IFN-dependent M2 macrophages polarization (114). The reason for these diametrically opposite results might be that the cGAS-STING signal pathway also influenced other downstream signal cascades, and one of the main factors was STAT6. PGC-1β, a coactivator of STAT6, could induce the polarization of M2 macrophages (136), which demonstrated that the STAT6 signal pathway played a crucial role in polarizing macrophages into M2 phenotype. Parallel to that, related studies confirmed that the STING signal pathway could indeed effectively activate the STAT6 (59, 137), which further demonstrated the complexity of the cGAS-STING signal pathway in regulating macrophages polarization. Similarly, considerable studies have found that the activation of the cGAS-STING signal pathway could not only promote the related effector T cells differentiation but also inhibit the related effector T cells differentiation under certain conditions. The type I IFN induced by the activated cGAS-STING signal pathway could up-regulate PD-L1 and IDO-1 expression, which would facilitate the production of suppressive Treg cells to inhibit the activation and differentiation of related effector T cells, thereby restraining the effective host immune responses to aggravate the severity of related diseases (70–72, 84, 103, 113). These seemingly contradictory results in different disease models suggest the complexity of the cGAS-STING signal pathway in regulating macrophages polarization and T lymphocytes differentiation, which also provides considerable prospects and directions for future research.

Based on the knowledge obtained from most studies, it is clear that we could develop various novel disease immunotherapeutic strategies targeting the cGAS-STING signal pathway. In cancer, the intrinsic anti-tumor immune efficiency of the cGAS-STING signal pathway inspires intense efforts to boost anti-tumor immunity in next-generation cancer immunotherapies by developing cGAS-STING signal pathway agonists. Meanwhile, the combination of the cGAS-STING signal pathway agonists and different cancer treatments (radiotherapy, chemotherapy) is also a promising option. However, considering that the activation of the cGAS-STING signal pathway is also associated with tumor metastasis, the possible side effects of agonists must be considered when using agonists to treat cancer. When using combination therapy, we should maximize the positive effects of combination therapy and minimize the negative effects. In autoimmune and inflammatory diseases, the important and valuable task is to develop efficient cGAS-STING signal pathway antagonist or gene knockout technology to alleviate the severity of the diseases. In terms of vaccine application, we confidently believe that STING agonists have great potential to aid in the development of new vaccines to prevent and treat infectious diseases such as HIV/AIDS, tuberculosis, and malaria. In the context of microbial and parasitic infections, the definite regulatory mechanism and immune function of the cGAS-STING signal pathway remain unknown. Therefore, it is necessary to further explore the knowledge in this area so that we can better develop relevant and effective drugs and treatments. In recent years, the studies on the cGAS-STING signal pathway have mainly remained at the animal and cell levels. In this process from animals to humans and even clinical application, there is still a large space and journey for us to explore. To sum up, the current research on the cGAS-STING signal pathway is only the tip of the iceberg. There are still many puzzles and problems in the study of the cGAS-STING signal pathway, and all aspects of knowledge are worthy of in-depth study and will be full of great prospects.

## Author Contributions

LO drafted and edited the manuscript, and prepared the figures. LO, AZ, and YXC collected relevant literature resources. YC decided on the topics, reviewed the manuscript, and instructed the manuscript completion. All authors contributed to the article and approved the submitted version.

## Funding

This work was supported by the National Natural Science Foundation of China (No.82073499, NO. 81773376).

## Conflict of Interest

The authors declare that the research was conducted in the absence of any commercial or financial relationships that could be construed as a potential conflict of interest.

## Publisher’s Note

All claims expressed in this article are solely those of the authors and do not necessarily represent those of their affiliated organizations, or those of the publisher, the editors and the reviewers. Any product that may be evaluated in this article, or claim that may be made by its manufacturer, is not guaranteed or endorsed by the publisher.

## Abbreviations

cGAS, cyclic guanosine monophosphate-adenosine monophosphate synthase; STING, stimulating interferon gene; cGAMP, cyclic guanosine monophosphate-adenosine monophosphate; type I IFN, type I interferon; APCs, antigen-presenting cells; Th, T helper; Treg, regulate T; TAMs, tumor- associated macrophages; DCs, dendritic cells; LAP, LC3-associated phagocytosis; TILs, tumor infiltrating lymphocytes; CDNs, cyclic dinucleotides; c-di-AMP, cyclic dimeric adenylate monophosphate; c-di-GMP, cyclic dimeric guanosine monophosphate; PD-L1, programmed death-ligand 1; Mtb, Mycobacterium tuberculosis; IFN, interferon.
